# Tim-3 protects against cisplatin nephrotoxicity by inhibiting NF-κB-mediated inflammation

**DOI:** 10.1038/s41420-023-01519-6

**Published:** 2023-07-01

**Authors:** Peiyao Li, Xuemiao Li, Wenbin Wu, Mengjia Hou, Guanyi Yin, Zhonghang Wang, Ziyu Du, Yuanfang Ma, Qiang Lou, Yinxiang Wei

**Affiliations:** Joint National Laboratory for Antibody Drug Engineering, The First Affiliated Hospital of Henan University, Henan University, Kaifeng, 475004 P.R. China

**Keywords:** Acute kidney injury, Apoptosis

## Abstract

The impact of Tim-3 (T cell immunoglobulin and mucin domain-containing protein 3) on cisplatin-induced acute kidney injury was investigated in this study. Cisplatin-induced Tim-3 expression in mice kidney tissues and proximal tubule-derived BUMPT cells in a time-dependent manner. Compared with wild-type mice, Tim-3 knockout mice have higher levels of serum creatinine and urea nitrogen, enhanced TUNEL staining signals, more severe 8-OHdG (8-hydroxy-2’ -deoxyguanosine) accumulation, and increased cleavage of caspase 3. The purified soluble Tim-3 (sTim-3) protein was used to intervene in cisplatin-stimulated BUMPT cells by competitively binding to the Tim-3 ligand. sTim-3 obviously increased the cisplatin-induced cell apoptosis. Under cisplatin treatment conditions, Tim-3 knockout or sTim-3 promoted the expression of TNF-α (tumor necrosis factor-alpha) and IL-1β (Interleukin-1 beta) and inhibited the expression of IL-10 (interleukin-10). NF-κB (nuclear factor kappa light chain enhancer of activated B cells) P65 inhibitor PDTC or TPCA1 lowed the increased levels of creatinine and BUN (blood urea nitrogen) in cisplatin-treated Tim-3 knockout mice serum and the increased cleavage of caspase 3 in sTim-3 and cisplatin-treated BUMPT cells. Moreover, sTim-3 enhanced mitochondrial oxidative stress in cisplatin-induced BUMPT cells, which can be mitigated by PDTC. These data indicate that Tim-3 may protect against renal injury by inhibiting NF-κB-mediated inflammation and oxidative stress.

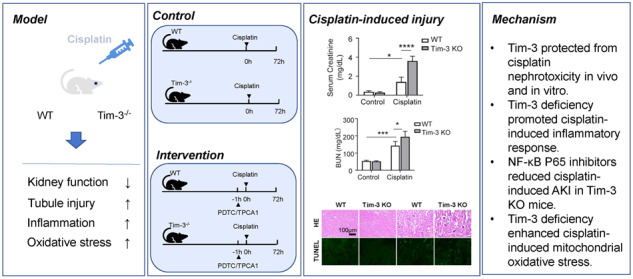

## Introduction

Acute kidney injury (AKI) is a frequent clinical diagnosis featured by a sudden decrease in kidney function [1], which is often accompanied by complex immune pathophysiological responses [2, 3]. Cisplatin is widely applied to chemotherapy for cancer. However, its application is limited usually by cisplatin nephrotoxicity. Cisplatin is not only cytotoxic, but also triggers an inflammatory cascade response leading to AKI [4, 5]. Therefore, it’s quite meaningful to study the molecular regulatory mechanism of cisplatin nephrotoxicity.

T cell immunoglobulin and mucin domain-containing protein 3 (Tim-3) belongs to Tim immunoregulatory protein family. Tim-3 is a negative regulator of immune or inflammatory response and is extensively expressed in various types of immunocytes [6]. Aside from its association with hematological [7] and autoimmune diseases [8] and regulation of immune responses in cancer [9, 10], Tim-3 was closely associated with the prevalence of kidney diseases [11, 12]. Cisplatin was reported to increase Tim-3 expression on CD8-positive T cells, which in turn led to the alleviation of peripheral neuropathy caused by chemotherapy [13].

Monoclonal anti-Tim-3 antibody was reported to be protective in the ischemia/reperfusion injury mice model [14]. In a clinical case with *Mycobacterium tuberculosis* infection, the high Tim-3 expression in CD3-positive circulating T cells was related to the progression of inflammatory kidney injury [15]. High expression of Tim-3 and low expression of Heme oxygenase-1 (HO-1) were also found in skin tissue from SARS-CoV-2 positive patients [16]. The level of NF-κB p65 phosphorylation was reduced after Tim-3 overexpression in lipopolysaccharide-induced orbital fibroblasts [17]. NF-κB activation inhibitor JSH-23 obviously reduced cisplatin-induced kidney injury and the expression of IL-10, IFN-γ, CCL2, and TNF-α [18]. However, the location, expression, function, and underlying regulatory mechanism of Tim-3 in cisplatin-induced AKI were not fully evaluated. The purpose of this research was to explore the impact of Tim-3 on cisplatin-induced AKI in vivo and in vitro, and whether Tim-3 played a role in protecting AKI through regulating NF-κB signaling pathway.

## Results

### Tim-3 deficiency aggravated cisplatin nephrotoxicity in mice model

It was well-known that Tim-3 was expressed mainly on different immunocytes such as T cells, macrophages, and so on. It is still not known whether Tim-3 is expressed in different organ tissues. Proteins were extracted from the kidney, liver, spleen, and lung tissues, respectively. Western blot analysis showed the obvious Tim-3 expression in the kidney (Supplemental Fig. 1a), indicating different Tim-3 expression patterns exist in different tissues. Moreover, there was no obvious Tim-3 expression difference between the kidney medulla and kidney cortex (Supplemental Fig. 1b). Tim-3 expression in mice kidney was induced by cisplatin on days 1, 2, and 3 (Supplemental Fig. 1c). Moreover, Tim-3 can be co-localized with LTL (Lotus Tetragonolobus Lectin) or DBA (Dolichos Biflorus Agglutinin), which were markers of the renal proximal tubule and collecting ducts, respectively (Supplemental Fig. 1d), suggesting that Tim-3 was expressed in the renal proximal tubule and collecting ducts.

**Fig. 1:**
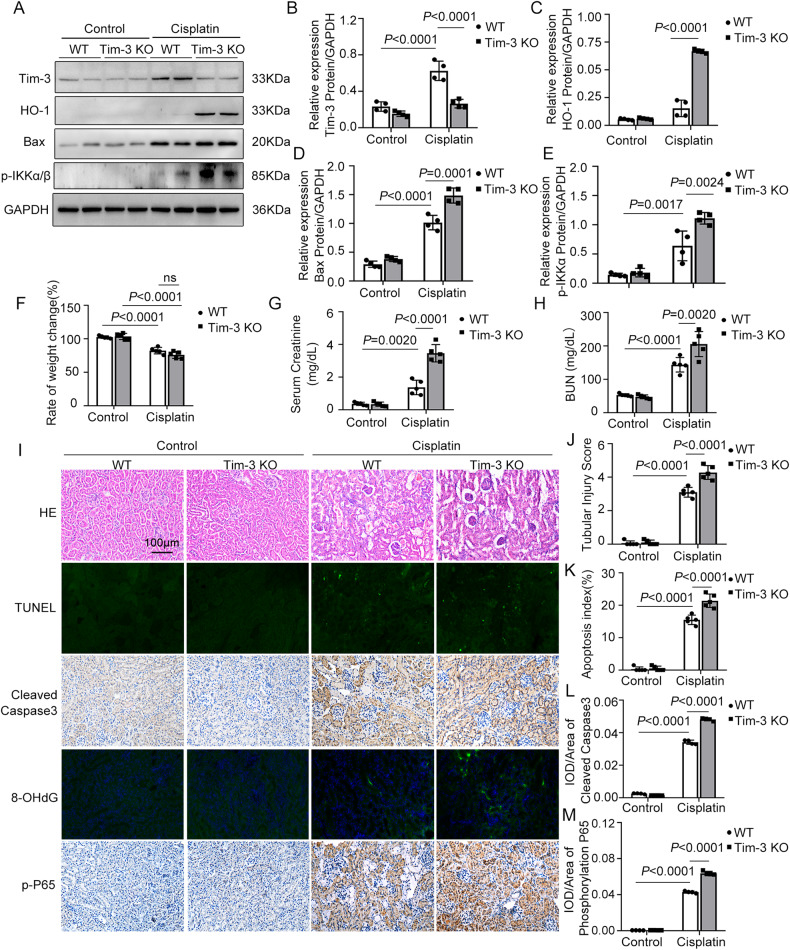
Tim-3 deficiency exaggerated cisplatin nephrotoxicity. C57/BL6 wildtype (WT) and Tim-3 knockout (KO) mice were stimulated with cisplatin (30 mg/kg) for 72 h. **A** The expression of Tim-3, HO-1, Bax, and phosphor-IKKα/β were determined by western blot analysis of three biological replicates. GAPDH was used as the internal control. **B**–**E** The densities of western blot bands were quantified using ImageJ software. **F** Ratios of body weight change. **G**, **H** Serum creatinine and BUN values of mice in different groups. **I** Hematoxylin and eosin (HE) staining and TUNEL staining of kidney cortex tissues from different groups of mice; Immunohistochemistry staining for cleaved caspase 3 and phosphor-p65 in the kidney cortex of cisplatin-treated WT or Tim-3-KO mice. Bar = 100 μm. **J** Tubular damage score was semi-quantified according to HE staining results. **K** Apoptosis index was the percentage of TUNEL-positive cells out of the total number of cells in the field. **L**, **M** Quantitative integrated optical density (IOD) analysis of the levels of cleaved caspase 3 and phosphor-p65 by immunohistochemical staining. Data represented as mean ± SD (*n* = 5 per group). Statistical significance was determined by a two-way ANOVA test.

In this study, the C57BL/6N mice was intraperitoneally injected with cisplatin to induce the cisplatin-AKI model. The Tim-3 expression was significantly induced by cisplatin treatment. After cisplatin stimulation, compared with wild-type (WT) mice, the expression of Tim-3 in Tim-3 KO mice decreased by 57.7% (*p* < 0.0001), and the expression of phosphor-IKKα/β (IκB kinase α/β) and oxidative stress-related proteins HO-1 and Bax (BCL2 Associated X) were increased by 6.1, 4.4, and 1.5 times, respectively (Fig. 1a–e). The Tim-3 mRNA level was also determined in Tim-3 WT and KO mice (supplemental Fig.1e). Quantitative real-time RT-PCR analysis indicated that the Tim-3 expression was upregulated by 1.9 times after cisplatin treatment. The Tim-3 mRNA level after Tim-3 knockout was nearly undetectable. Under the cisplatin condition, Tim-3 knockout decreased the Tim-3 expression by 88.6%.

After cisplatin treatment, the body weight of WT mice and Tim-3 KO mice decreased by 19 and 25%, respectively (Fig. 1f); compared with WT mice, creatinine and BUN levels in the serum of Tim-3 KO mice were significantly increased by 2.59 times and 1.36 times, respectively (Fig. 1g, h), suggesting that Tim-3 can reduce the damage degree of renal function in cisplatin-induced AKI mice without influencing body weight.

The renal damage degree was further evaluated by HE and TUNEL staining. HE staining of pathological sections of Tim-3 KO mice after cisplatin treatment showed a more disordered arrangement of renal tubules, dilation of the lumen, the disappearance of internal brush-like margin structure, shedding of some epithelial cells from the basement membrane, and rupture of basement membrane (Fig. 1i). After cisplatin treatment, the tubular injury score of Tim-3 KO mice was higher than that of WT mice (Fig. 1j). The fluorescence intensity of TUNEL staining in renal tissue sections of Tim-3 KO mice was much more than that of WT mice (Fig. 1k). The Tim-3 knockout mice exhibited more severe 8-OHdG (8-hydroxy-2’ -deoxyguanosine) accumulation than the WT mice (Fig. 1i). These data suggest that Tim-3 KO exacerbates cisplatin nephrotoxicity.

Then immunohistochemistry IHC staining was used to examine the extent of caspase 3 cleavage in renal tissues of mice in control and cisplatin groups. There was no obvious brown-positive area in the control group. In the cisplatin group, the caspase 3 cleavage was more obvious than in the control group. Compared with WT mice, cisplatin increased the level of cleavage of caspase 3 by 1.4 times than Tim-3 KO mice (Fig. 1l). In addition, the phosphorylated P65 protein expression was increased by 108.3 times after cisplatin treatment in WT mice. Tim-3 deficiency increased the expression of p-P65 by 1.49 times during cisplatin treatment (Fig. 1m). The results indicated that Tim-3 KO may increase the oxidative stress level and aggravate the damage degree of renal tissues by activating the NF-κB pathway.

### Tim-3 protected from cisplatin-induced apoptosis of kidney tubular epithelial cells

**Fig. 2:**
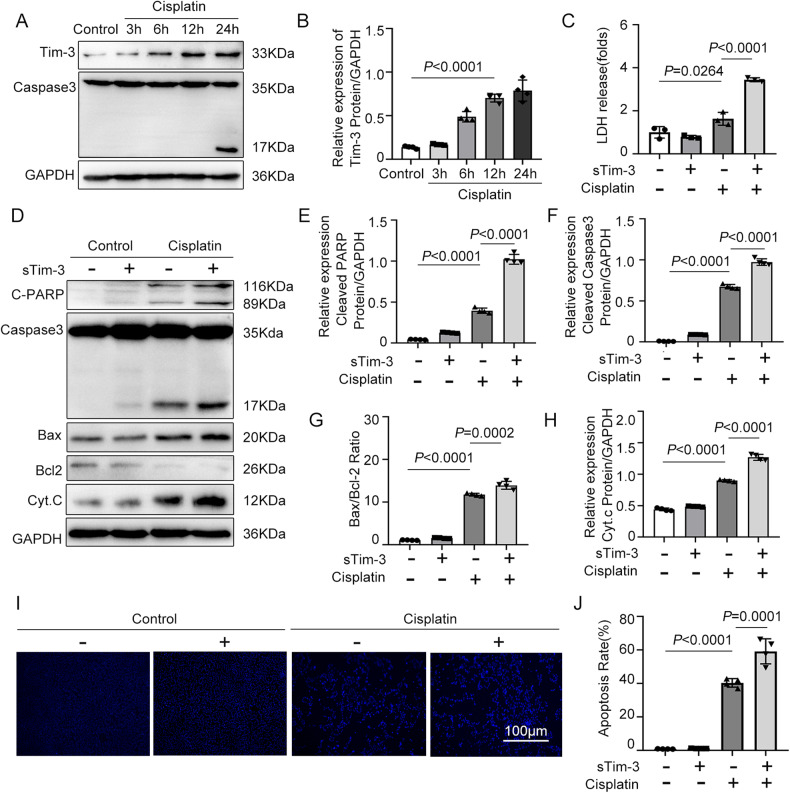
Soluble Tim-3 promoted cisplatin-induced apoptosis of kidney tubular epithelial cells. **A** BUMPT cells were stimulated with 40 μM cisplatin for 0, 3, 6, 12, and 24 h. Western blot analysis of Tim-3 and caspase 3. GAPDH was used as the internal control. **B** The densities of western blot bands were quantified using ImageJ software. **C** LDH levels in culture medium from BUMPT cells treated by 40 μM cisplatin with and without soluble Tim-3 recombinant protein. **D** Western blot analysis of the levels of cleaved PARP, cleaved caspase 3, Bax, Bcl-2, and Cytochrome C. GAPDH was used as the internal control. **E**–**H** The densities of western blot bands were quantified using ImageJ software. **I** Hoechst 33342 staining of BUMPT cells in different groups. **J** The apoptotic rate was calculated as the percentage of Hoechst-stained cells over the total number of cells in the field. Data represent mean ± SD (*n* = 3–4 per group). Statistical significance was determined by a one-way ANOVA test. The experiments were repeated at least three times.

The expression of Tim-3 in the cisplatin-induced cell apoptosis model was also investigated. BUMPT (Boston University mouse proximal tubular) cells were stimulated with 40 μM cisplatin for 0, 3, 6, 12, and 24 h. Tim-3 expression was upregulated with increasing treatment time (Fig. 2a, b).

To verify that Tim-3 is protective against cisplatin-induced renal tubular epithelial cell apoptosis, soluble Tim-3 (sTim-3) protein was purified and was used to intervene in cisplatin-stimulated BUMPT cells. The sTim-3 is a splice mutant that lacks mucin and transmembrane domains, can bind to Tim-3 ligand, and thus block its function. BUMPT cells (8 × 10^5^) were inoculated into 35 mm dishes. After treatment of BUMPT cells with 40 μM cisplatin with or without sTim-3 protein (80 μg/mL), sTim-3 obviously upregulated the levels of LDH (lactate dehydrogenase) (Fig. 2c). The apoptosis of BUMPT cells were also evaluated by western blot analysis of the levels of cleaved PARP, cleaved caspase 3, Bax and cytochrome C (Cyt. C) (Fig. 2d–h). Cisplatin stimulation obviously increased PARP cleavage, caspase 3 cleavage, and the expression of Bax and Cyt. C, and decreased Bcl-2 (B-cell lymphoma-2) expression. Compared with single cisplatin treatment group, the levels of cleaved caspase 3 and cleaved PARP were increased by 1.45 and 2.59 times, respectively, after sTim-3 intervention and cisplatin stimulation. Moreover, the BUMPT cells apoptosis were observed by staining with Hoechst 33342 solution (Fig. 2i, j).

### Tim-3 was involved in regulating the cisplatin-induced inflammatory response

**Fig. 3:**
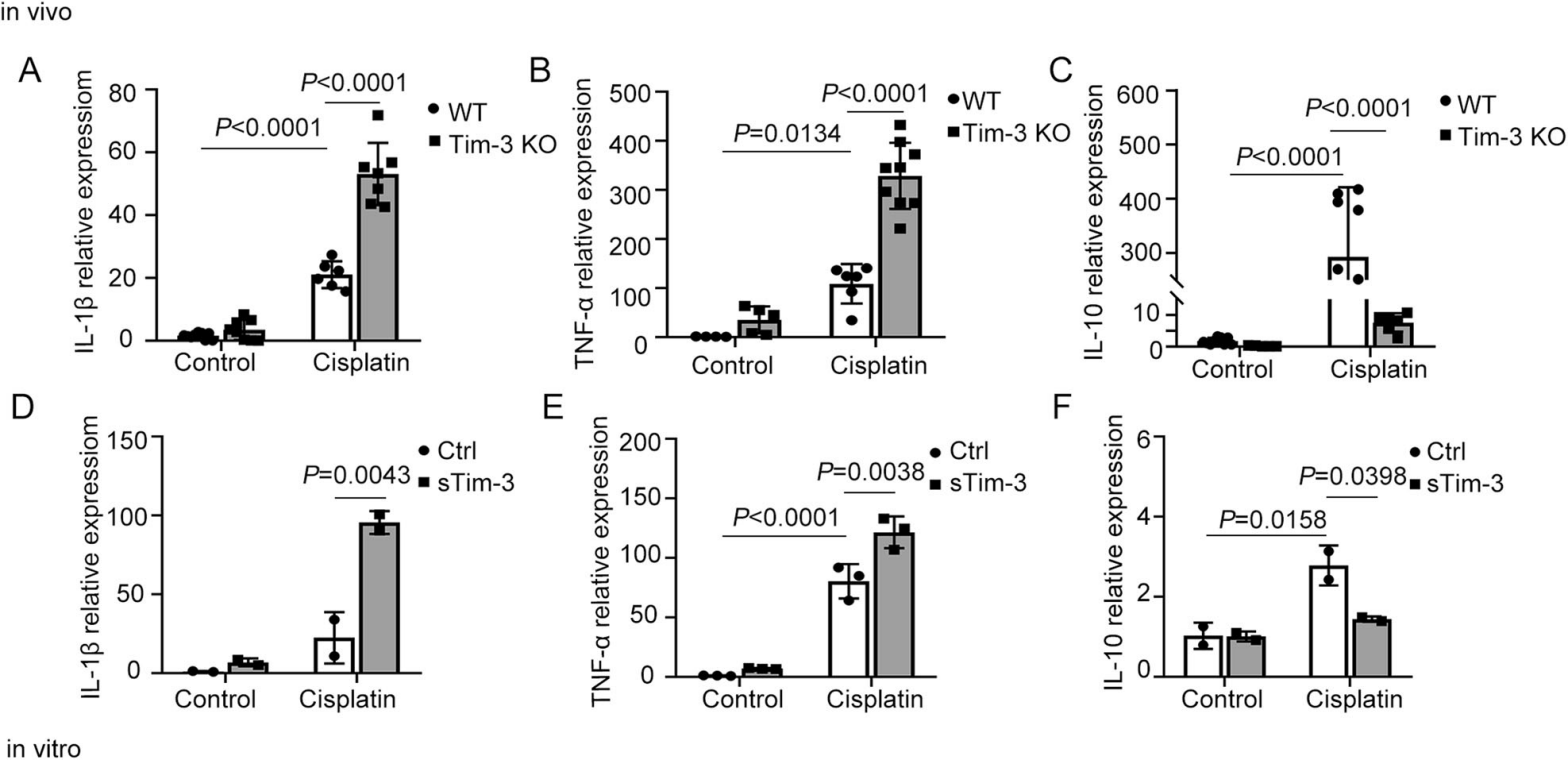
The effect of Tim-3 on the expression of inflammatory factors. **A**–**C** C57/BL6 wild-type and Tim-3 knockout mice were stimulated with cisplatin (30 mg/kg) for 72 h. IL-1β, TNF-α, and IL-10 mRNA levels were determined using quantitative RT-PCR (*n* = 3–5). **D**–**F** BUMPT cells were treated with 40 μM cisplatin with and without soluble Tim-3 recombinant protein for 24 h. IL-1β, TNF-α, and IL-10 mRNA levels were determined using quantitative RT-PCR (*n* = 3). Data were shown as means ± SD (*n* = 2–9 per group). Statistical significance was determined using two-way ANOVA followed by Tukey’s post hoc test. The experiments were repeated at least three times.

Inflammatory factors are key mediators leading to tissue damage. In order to clarify the level changes of inflammatory reaction in the Tim-3 KO AKI mouse model, the inflammation-related indicators were then detected. Renal cortex tissues of AKI mice models were isolated and the changes of inflammatory factors after Tim-3 KO were observed by real-time PCR. The expression of IL-1β, TNF-α, and IL-10 were increased after cisplatin treatment. The pro-inflammatory factors TNF-α and IL-1β were significantly upregulated by 2.53 times and 3.03 times in renal tissues of Tim-3 KO AKI mice, respectively (Fig. 3a, b). IL-10 was lowered by 97.4% after Tim-3 knockout (Fig. 3c).

The expression changes of inflammatory factors were also confirmed in BUMPT cells. Compared with the cisplatin group, co-stimulation of cisplatin and sTim-3 protein significantly upregulated pro-inflammatory factors IL-1β and TNF-α by 4.27 and 1.51 times (Fig. 3d, e) and downregulated anti-inflammatory factor IL-10 by 48.2%, respectively (Fig. 3f).

### NF-κB P65 inhibitors reduced cisplatin-induced AKI in Tim-3 KO mice

**Fig. 4:**
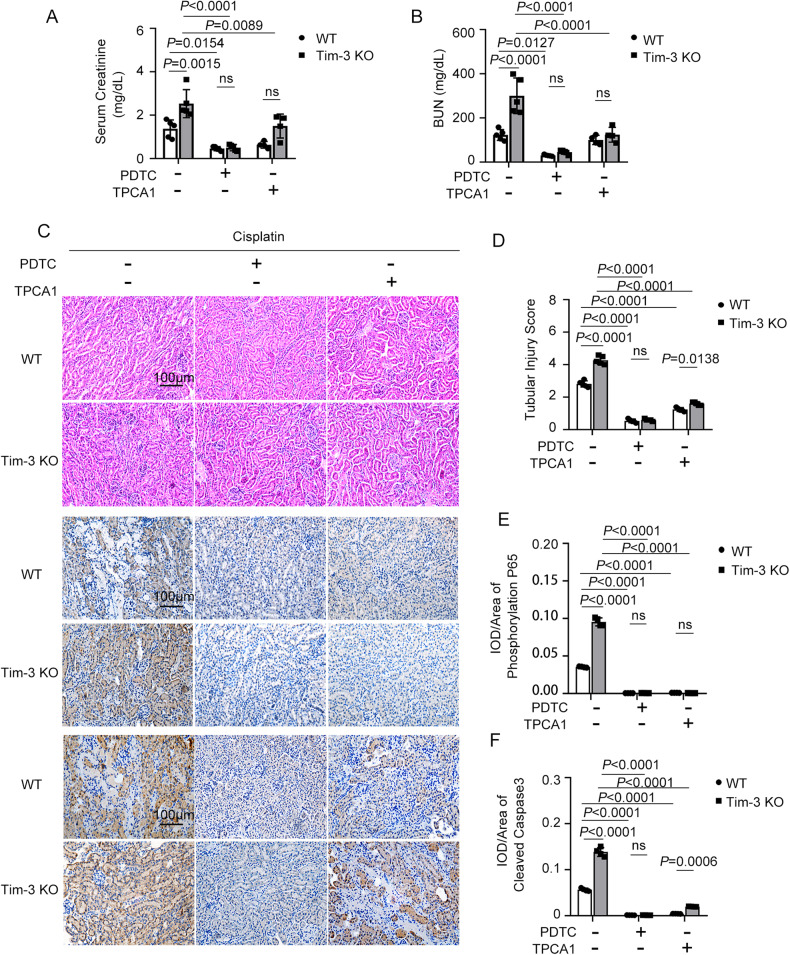
NF-κB inhibitors attenuated kidney injury in Tim-3 knockout mice. C57/BL6 wild-type and Tim-3 knockout mice were stimulated by cisplatin (30 mg/kg) with and without PDTC or TPCA1. **A**, **B** Serum creatinine and BUN values were determined. **C** Immunohistochemistry staining for phosphor-p65 and cleaved caspase 3 in the kidneys of different groups; Representative HE staining of kidney cortex tissues. Bar = 100 μm. **D** Quantitative IOD analysis of the level of phosphor-p65 by immunohistochemical staining. **E** Tubular damage score was semi-quantified according to HE staining results. **F** Quantitative IOD analysis of the level of cleaved caspase 3 by immunohistochemical staining. Data represent mean ± SD (*n* = 4–5 per group). Statistical significance was determined using two-way ANOVA followed by Tukey’s post hoc test. The experiments were repeated at least three times.

The pro-inflammatory response can be activated by the NF-κB complex [19]. To verify the key role of NF-κB activation in AKI induced by cisplatin and Tim-3 deficiency, the inhibitors of NF-κB P65, PDTC (pyrrolidine dithiocarbamate) and TPCA1 were applied in cisplatin-induced AKI mice models. After treatment with PDTC or TPCA1, serum creatinine in Tim-3 KO AKI mice were decreased by 80.2 and 40.8%, respectively (Fig. 4a); serum BUN in Tim-3 KO AKI mice was decreased by 85.0 and 58.8 %, respectively (Fig. 4b). HE staining showed that renal tubules of the AKI mice treated with NF-κB P65 inhibitor were arranged more closely and orderly, and the damage of the internal brush edge and lumen morphology were significantly alleviated (Fig. 4c, d). IHC staining was used to detect the levels of phosphorylated p65 and caspase 3 cleavage in the renal tissues of mice in each group. Compared with WT mice, more positive brown signals were detected in Tim-3 KO mice with cisplatin treatment (Fig. 4c, e, f). The p65 phosphorylation and caspase 3 cleavage in renal tissues of WT and Tim-3 KO AKI mice was reduced significantly after co-treatment of cisplatin and PDTC or TPCA1. For the AKI mice treated with NF-κB P65 inhibitor PDTC or TPCA1 only, there was basically no brown-positive expression area. These data suggest that NF-κB activation contributes to the aggravated cisplatin nephrotoxicity caused by Tim-3 KO.

### NF-κB P65 inhibitors decreased renal tubular cells apoptosis induced by cisplatin and sTim-3

**Fig. 5:**
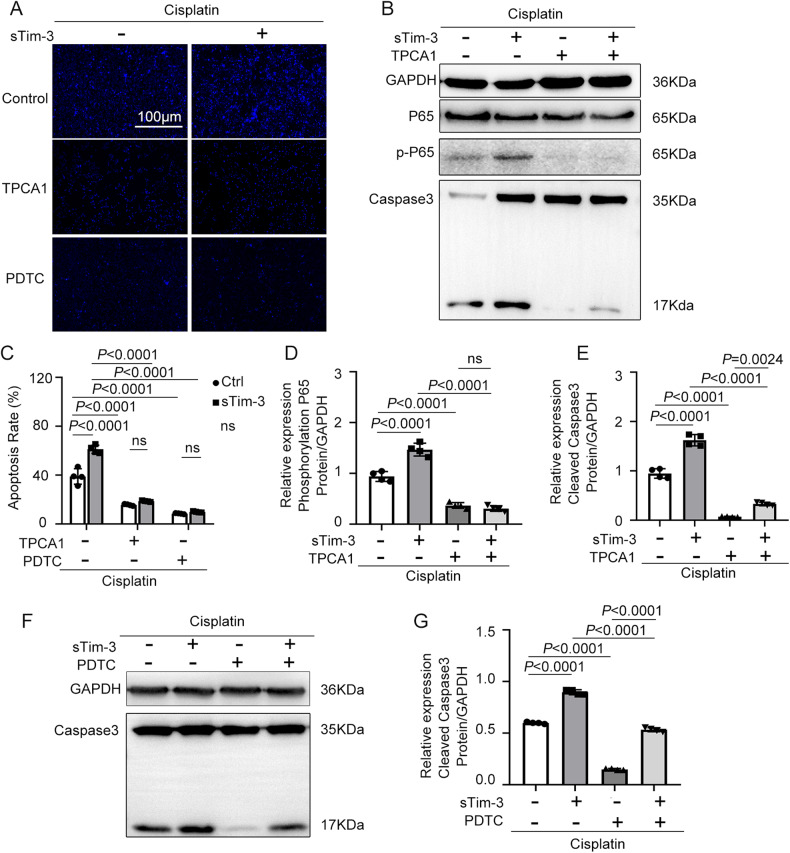
The effect of NF-κB inhibitors and sTim-3 on kidney tubular epithelial cells’ apoptosis. After pre-treatment with PDTC or TPCA1 for 1 h, BUMPT cells were stimulated by 40 μM cisplatin with and without sTim-3 for 24 h. **A** Hoechst 33342 staining of BUMPT cells in different groups. **B** The expression of P65, phosphor-p65, and cleaved caspase 3 were determined by western blot. GAPDH was used as the internal control. **C** The apoptotic rate was calculated as the percentage of Hoechst-stained cells over the total number of cells in the field. **D**, **E** The densities of western blot bands were quantified using ImageJ software. **F** Western blot analysis of the levels of cleaved caspase 3. GAPDH was used as the internal control. **G** The densities of western blot bands were quantified using ImageJ software. Data represent mean ± SD (*n* = 4 per group). Statistical significance was determined using two-way ANOVA (**C**) or one-way ANOVA (**D**, **E**, **G**) followed by Tukey’s post hoc test. The experiments were repeated at least three times.

BUMPT nuclei were stained with Hoechst to observe cell apoptosis. Cisplatin and sTim-3 treatment significantly increased cisplatin-induced cell apoptosis. After treatment with NF-κB inhibitors TPCA1 and PDTC, the fluorescence intensity decreased significantly (Fig. 5a, c). BUMPT cells were treated with cisplatin and sTim-3, and P65 phosphorylation and caspase 3 cleavage were measured using Western blot. Results showed that NF-κB inhibitors TPCA1 and PDTC could significantly reduce the levels of P65 phosphorylation and cleavage of caspase 3 in BUMPT cells treated with cisplatin and sTim-3 (Fig. 5b, d–g). These data suggest that Tim-3 protects cisplatin-induced renal tubular cell apoptosis through the NF-κB pathway.

### Tim-3 deficiency enhanced cisplatin-induced mitochondrial oxidative stress

**Fig. 6:**
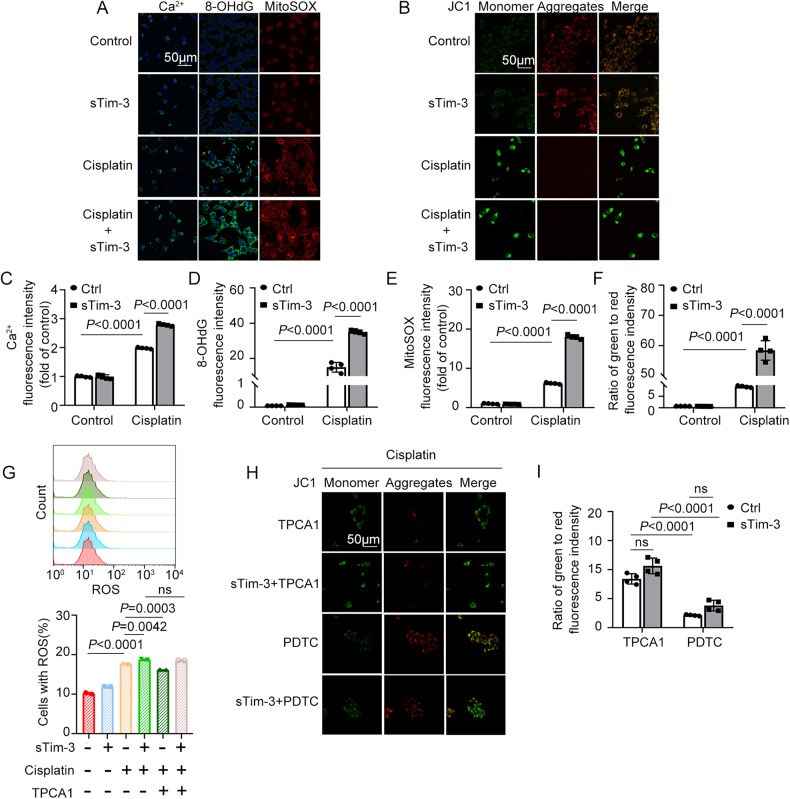
The effect of sTim-3 and NF-κB inhibitors on cisplatin-induced mitochondrial oxidative stress in BUMPT cells. After pre-treatment with PDTC or TPCA1 for 1 h, BUMPT cells were stimulated by 40 μM cisplatin with and without sTim-3 for 24 h. **A** Immunofluorescence analysis of calcium (green), 8-OHdG (green), and mitochondrial superoxide (MitoSOX) (red). The nuclei were labeled with DAPI (4′,6-diamidino-2-phenylindole, blue). Bar = 50 μm. **B** Mitochondrial membrane potential was visualized by the bright red fluorescence signals (JC-1 aggregates) and green signals (JC-1 monomer). Bar = 50 μm. **C**–**F** The fluorescence intensity of calcium, 8-OHdG, MitoSOX, and JC-1 were quantified using ImageJ software. **G** Measurement of total cellular reactive oxygen species (ROS) by flow cytometry; data from three independent experiments in BUMPT cells were processed by the GraphPad Prism software. **H** The mitochondrial membrane potential was visualized by JC-1 staining. Bar = 50 μm. **I** The fluorescence intensity of JC-1 was quantified by ImageJ software. Data represent mean ± SD (*n* = 4 per group). Statistical significance was determined using one-way ANOVA (**G**) or two-way ANOVA (**C**–**F**, **I**) followed by Tukey’s post hoc test. The experiments were repeated at least three times.

Excessive levels of ROS (reactive oxygen species) promote pathological development associated with renal injury. Mitochondrial calcium overload can lead to ROS increase. The inhibition of Tim-3 signaling increased cytoplasmic calcium overload and mitochondrial ROS levels (MitoSOX) in cisplatin-induced BUMPT cells (Fig. 6a, c, e). The fluorescence intensity of oxidative stress marker 8-OHdG in BUMPT cells treated with sTim-3 and cisplatin was significantly increased by 2.33 times compared with that in the cisplatin group (Fig. 6a, d). In addition, sTim-3 treatment significantly downregulated mitochondrial membrane potential as indicated by the declined bright red signals (JC-1 aggregates) and enhanced green signals (JC-1 monomer) (Fig. 6b, f).

To examine the roles of NF-κB signals in the process of sTim-3 up-regulating oxidative stress, flow cytometry assay and immunofluorescence staining were performed to analyze the ROS release and mitochondrial membrane potential changes, respectively. Here, we found that cisplatin treatment increased ROS levels in BUMPT cells, which can be inhibited by NF-κb P65 inhibitor TPCA1. Compared with the cisplatin group, the blockade of Tim-3 increased ROS release in BUMPT cells treated with cisplatin by 1.06 times. However, the effects of ROS levels induction by Tim-3 blocking in cisplatin-stimulated BUMPT cells were not affected by TPCA treatment (Fig. 6g), indicating that there are other factors involved in the process of sTim-3 inducing ROS release. Furthermore, the other NF-κB inhibitor PDTC with significant antioxidant properties increased the bright red fluorescence signals of BUMPT cells, indicating a significant increase of mitochondrial membrane potential. In contrast, TPCA only slightly increased the bright red fluorescence signals (Fig. 6h, i). These results suggest that Tim-3 negatively regulates oxidative stress and may protect against renal injury by inhibiting mitochondrial oxidative stress in renal tubular epithelial cells.

## Discussion

In the present study, the role of Tim-3 in the renal pathogenesis of cisplatin nephrotoxicity was investigated. Our findings indicate that Tim-3 protects against AKI through the NF-κB molecular pathway. The proximal tubular injury was aggravated after Tim-3 knockout, which can be partly restored by inhibition of NF-κB activation. Tim-3 could be a diagnostic marker of cisplatin AKI and a potential target for alleviating renal injury and slowing down its development.

Tim-3 expression was induced after cisplatin treatment at an early time point. Tim-3 may be used as an effective molecular marker for monitoring the progress of acute kidney injury in those patients who receive cisplatin chemotherapy. Tim-3 was also found to be induced in other kidney injury models. In the renal ischemia-reperfusion injury mice model, Tim-3 and TLR4 (Toll-like receptor 4) expression were reported to be increased in macrophages [20]. In patients with IgA nephropathy [21] or systemic lupus erythematosus nephropathy [22], a high level of Tim-3 was detected in kidney tissues. The sTim-3 in serum was highly expressed and was very sensitive for the diagnosis of end-stage kidney disease [23]. Thus, Tim-3 may have important value in the diagnosis of acute and chronic kidney disease.

In the renal ischemia-reperfusion injury (IRI) mice model, Tim-3 expression were reported to be increased in macrophages. In addition, the macrophages with high expression of Tim-3 indeed played protective roles in ischemic reperfusion kidney injury [20]. However, in the cisplatin nephrotoxicity model, there is nearly no systematic research about the protective mechanism of Tim-3. In this study, we found Tim-3 protected against cisplatin nephrotoxicity by inhibiting NF-κB-mediated inflammation. Immune cells such as T cells and NK cells with high expression of Tim-3 may also be involved in the protective effects of Tim-3 on cisplatin-induced nephrotoxicity. In order to investigate the effect of macrophages with high expression of Tim-3 on the function of renal tubular epithelial cells, bone marrow-derived macrophages (BMDMs) were isolated from WT and Tim-3 KO mice. The BMDMs were prepared as previously described [24]. Using the real-time RT-PCR method, we observed high expression of Tim-3 in BMDMs from WT mice, and nearly no expression in BMDMs from Tim-3 KO mice (Supplemental Fig. 4A). The supernatants from BMDMs were further used to treat BUMPT cells for 12 h. Western blot results indicated that BMDMs supernatants from Tim-3 KO mice significantly promoted caspase 3 cleavage, compared with that from WT mice (Supplemental Fig. 4B). Thus, macrophages with high expression of Tim-3 may contribute to the protective effects of Tim-3 on cisplatin-induced nephrotoxicity.

On one hand, cisplatin induced nephrotoxicity, but on the other hand cisplatin also induced Tim-3 expression to inhibit the inflammation. There may be a negative feedback regulatory system existing in cisplatin condition, that is, Tim-3 was induced to fight against kidney injury at early time points, but failed to impede its development. This scenario was also found in other proteins. In our previous study, during cisplatin nephrotoxicity, we found the activation of factors such as Hsf1 (heat shock factor 1) [25] and Hnf1β (hepatocyte nuclear factor 1β) [26] manifested by increased expression and nuclear translocation. The two transcription factors both played protective roles in cisplatin-induced AKI.

Tim-3 deficiency in mice or blocking in proximal renal tubular epithelial cells leads to high levels of IL-1β and TNF-α and low levels of IL-10, indicating that Tim-3 may protect from cisplatin nephrotoxicity by inhibiting immune response. This conclusion was consistent with that of the kidney IRI model reported by Sho Hasegawa et al. [27]. During kidney IRI, Tim-3-positive macrophages were accumulated in kidney tissue, exhibited anti-inflammatory phenotypes, and contributed to protection from kidney injury. Tim-3 was reported to positively regulate TLR4 expression in Raw 264.7 cells [28]. In endosomes, TLR4 can induce the type I interferon IFN-β, IL-10, and other cytokines [29]. Thus, TLR4 may be a mediator in the process of Tim-3 inhibiting inflammation. However, Tim-3 signaling also inhibited TLR4-mediated NF-κB activation in the acute liver injury model [30]. Moreover, Tim-3 may be the downstream protein of TLR4, because Tim-3 affected macrophage polarization in TLR4 dependent way [31]. It’s worthwhile to further investigate whether Tim-3 regulating NF-κB activity is dependent on TLR4 in the cisplatin-induced AKI model.

The soluble extracellular region of Tim-3 could be shed into serum and regulate autoimmunity [32]. Tim-3 ablation alone did not promote autoimmune response and was inclined to regulate T-cell function synergistically with other immune inhibitory molecules such as PD-1 or CTLA4 [33]. Thus, the combined administration of Tim-3 and PD-1 may provide a new approach to protection against acute kidney injury.

TIM-3 can bind to its ligand HMGB1 (High mobility group box 1) [34]. HMGB1 was reported to contribute to increased cell apoptosis and was sensitive to oxidative stress [34]. These findings were consistent with the current results that HMGB1 expression was increased during cisplatin treatment and was further upregulated in cisplatin-stimulated Tim-3 knockout mice (supplemental Fig. 3). HMGB1 may act as an upstream component of Tim-3 signaling and Tim-3 negatively regulate HMGB1 expression during cisplatin nephrotoxicity.

Cisplatin treatment caused a progressive decrease in mice’s body weight. However, after cisplatin treatment, there was no obvious difference in the body weight between Tim-3 deficiency and WT mice. However, the expression of pro-apoptotic molecule caspase 3 splice, PRAP splice, and Bax increased after sTim-3 intervention, while the anti-apoptotic molecule Bcl-2 expression decreased significantly. Bax and Bak can be oligomerized at mitochondrial out membrane, and are important regulators of mitochondrial integrity and cell apoptosis [35]. Therefore, Tim-3 may regulate kidney injury and proximal tubule cell apoptosis through intrinsic apoptotic pathways.

NF-κB P65 is a critical transcription factor regulating the inflammatory response. In this study, NF-κB P65 inhibitor PDTC reduced more obvious acute kidney injury than TPCA in cisplatin-treated Tim-3 KO mice. This may be caused by the different targets of these inhibitors. PDTC targets NFκB, while TPCA targets the upstream IKKβ signaling [36]. Moreover, PDTC is not only a potent inhibitor of NF-κB, but also acts as an antioxidant against the toxic effects of free radicals and interferes with inflammatory cytokines production. The oxidative stress level changes may contribute to the cisplatin nephrotoxicity of Tim-3 KO mice.

Oxidative stress is a key factor promoting the progression of AKI. ROS can play an important role as an important mediator in activating pro-inflammatory signals, and can lead to the degradation of mtDNA. In the present study, we found that Tim-3 negatively regulates the ROS levels in cisplatin-treated BUMPT cells, as indicated by increased cytoplasmic calcium overload, increased mitochondrial membrane potential, and enhanced expression of oxidative stress marker 8-OHdG. There are several other similar reports about the negative regulation of ROS production by Tim-3. Tim-3 deficiency in nonalcoholic steatohepatitis mice contributed to enhanced production of ROS, IL-1β, and IL-18 [37]. Tim-3 deletion increases ROS production and inflammasome activation in dendritic cells [38].

ROS and NF-κB activation can regulate each other in a context-dependent manner, that is, ROS can lead to NF-κB activation or repression and NF-κB can also alleviate or exacerbate oxidative stress [39]. Interestingly, NF-κB inhibitor TPCA only slightly affect the ROS production in Tim-3 knockout mice, while PDTC significantly enhanced ROS release. This further suggests that oxidative stress contributes to the AKI progress caused by cisplatin and Tim-3 signal blocking.

**Fig. 7:**
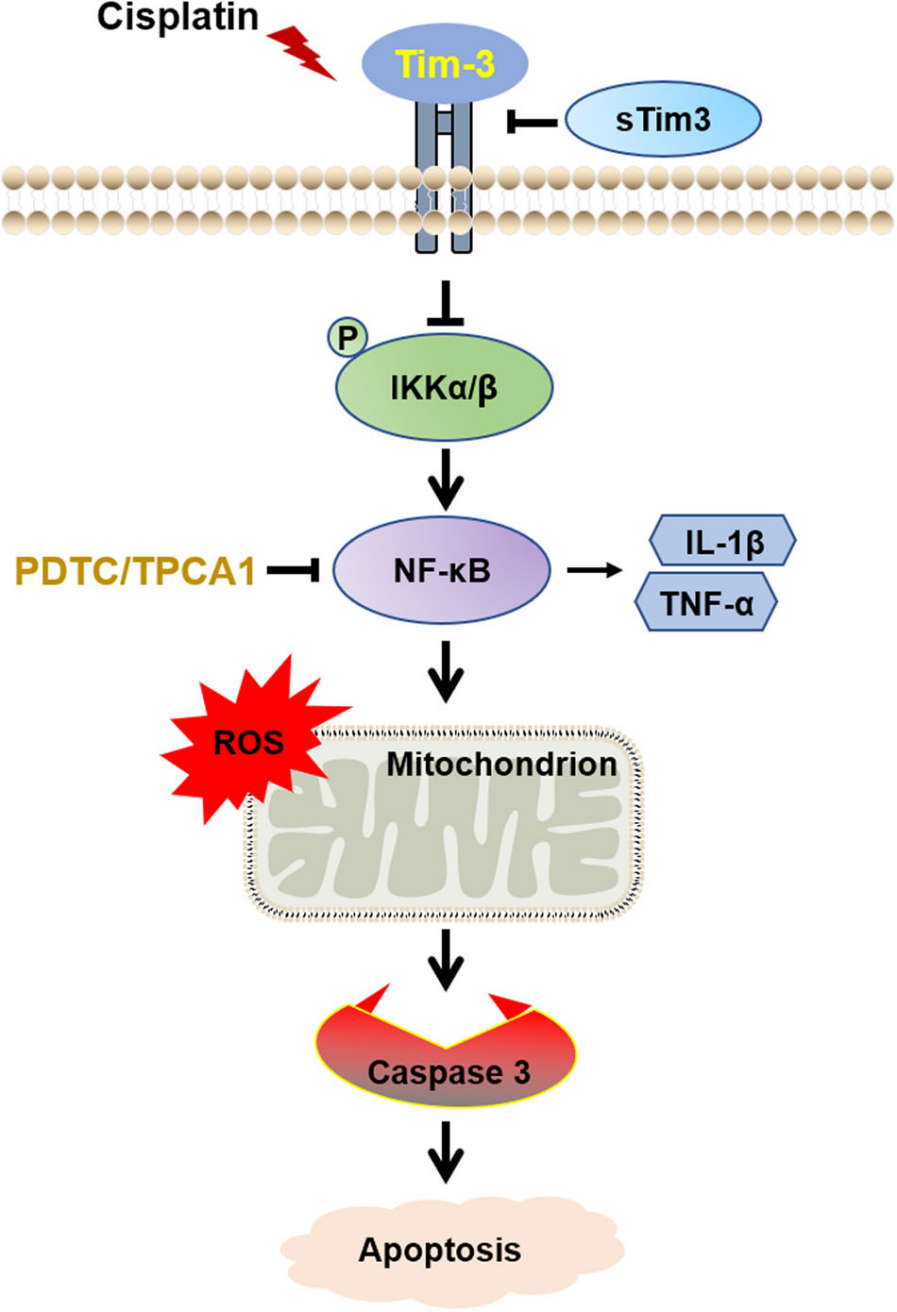
Schematic diagram of protection mechanism of Tim-3 in cisplatin-induced acute kidney injury. Tim-3 expression can be induced during cisplatin nephrotoxicity, functions to alleviate mitochondrial oxidative stress and cell apoptosis by inhibiting NF-kB signaling pathway.

In summary, Tim-3 expression can be induced during cisplatin nephrotoxicity, and functions to alleviate mitochondrial oxidative stress and cell apoptosis by inhibiting the NF-kB signaling pathway (Fig. 7). The limitation of the current study may be that the cisplatin-induced AKI mice model does not faithfully mimic human disease status. The expression and protective role of Tim-3 during cisplatin treatment need to be further confirmed in the human condition.

## Materials and methods

### Animal design

Tim-3 knockout mice was generated as previously described [40]. Briefly, Havcr2f/f and EIIa-cre mice (C57BL/6) were obtained from Dr. Gencheng Han (Institute of Basic Medical Sciences, China). Tim-3-flox mice were mated with EIIa-cre mice to generate Tim-3 KO (Havcr2f/f) mice (supplemental Fig.1f). The genotyping assays were conducted by PCR of the tail venous serum of knockout mice (supplemental Fig. 1g). The primers for genotyping were listed in Supplemental Table 1.

Six to eight weeks old wildtype or Tim-3 knockout male C57BL/6 mice were housed under temperatures of about 20 ^o^C and 40–60% humidity, and were exposed to a standard light/dark cycle. Five mice were included in each group to meet the minimum sample size requirement to perform one-way or two-way ANOVA analysis. Double-blind studies of the effects of NF-κb P65 inhibitors were conducted on randomly grouped wildtype or Tim-3 knockout mice. These mice were randomly grouped and were injected with PDTC (50 mg/kg, intraperitoneal injection) or TPCA1 (1 mg/kg, tail vein injection). One hour later, the mice were administered 30 mg/kg cisplatin by intraperitoneal injection. After 3 days, mice were anesthetized and sacrificed. The cervical venous serum and kidney cortex were collected for further analysis. The approval for animal experiments were obtained from the Animal Experiment Ethics Committee of Henan University (No. DWLL20200102).

### Renal function determination

The whole blood samples were remained for about 30 min and then were properly centrifuged (10 min, 500×*g*) to collect serum. Creatinine (0420-500, Stanbio Laboratory) and BUN (0580-250, Stanbio Laboratory) levels in serum were determined using commercial kits.

### Histopathologic examination

The kidney cortex sections were embedded with paraffin and serially cut with a thickness of 3 μm. The sections were dewaxed in xylene, subjected to ethanol with different concentrations from 100 to 70% (v/v), rehydrated with water for 10 min, and then were stained with hematoxylin (181220, Shanghai Jinsui company, China) and eosin (180109, Shanghai Jinsui company, China). After HE staining, the pathological sections were photographed using a microscope (Olympus IX53).

### TUNEL staining

The kidney cortex tissues were dewaxed, rehydrated, and permeabilized with Proteinase K and treated with reaction buffer (Vazyme A112) at 37 °C for 60 min, and then counterstained with Hoechst 33342 (10 μg/mL; Beyotime company, China) for 5 min. The TUNEL-stained positive signals were observed under a fluorescence microscope (Nikon A1R Storm).

### Immunohistochemical staining

The kidney cortex tissues were dewaxed, rehydrated, and incubated with 3% H_2_O_2_ at room temperature and protected from light for 20 min. Anti-cleaved caspase 3 antibody (9664, CST) and anti-phospho-NF-κB p65 (Ser536) antibody (Abcam ab86299, Abcam) were incubated with the sections overnight at 4 °C followed by incubation with blocking solution (5% BSA) for 30 min. The next day, the secondary antibodies (SA1022, Wuhan Boster biological company, China) were used to incubate with the sections for about 1 h at room temperature. After DAB (ZLI-9018, Beijing Zsbio company, China) staining (brown) and counterstaining with hematoxylin (blue), the staining effects were observed using an optical microscope (Olympus IX53) and analyzed by Image-Pro Plus software.

### Histoimmunofluorescence staining

After dewaxing, rehydration, and blocking with BSA, the kidney cortex sections were treated with anti-8-OHdG antibody (Santa Cruz company, sc-66036) overnight at 4 °C. Then, the Alexa Fluor 488–labeled secondary antibody (A-11001, Thermo Fisher Scientific) was used. For detection of co-localization of Tim-3 and LTL/DBA, the primary antibodies were anti-Tim-3 antibody (1:100, ab185703, Abcam), anti-LTL antibody (1:200, FL-1321-2, Vector Laboratories), and anti-DBA antibody (1:100, FL-1031, Vector Laboratories), the secondary antibody was IgG antibody conjugated with Alexa Fluor 594 dye (1:1000, A21207, Thermo Fisher Scientific). After DAPI staining of nuclei, the sections were photographed under a fluorescence microscope (Nikon A1R Storm).

### Preparation of soluble Tim-3 protein

Soluble Tim-3 (sTim-3) is a 35 kd extracellular domain protein that blocks Tim-3 activity by competitively binding to the Tim-3 ligand [41]. Stim-3 recombinant protein was produced as described elsewhere [42]. Briefly, the extracellular region (134–643 bp) of Tim-3 was amplified by PCR from mouse kidney tissue. The primers used for PCR amplification of the extracellular domain of Tim-3 were listed in Supplemental Table 2. The amplified Tim-3 gene and pET32a plasmid were double-digested with XhoI/BamHI. The Tim-3 extracellular domain and linear vector with a 6× histidine tag were linked together to construct recombinant pET32a-Tim-3 plasmid (supplemental Fig.2a). Pet32a-Tim-3 plasmid was transformed into *Escherichia coli* BL21 competent cells. After IPTG induction, the bacteria were ultrasonicated. After centrifugation, the supernatant was purified to obtain soluble Tim-3 protein. The purity of sTim-3 was examined using SDS–PAGE (supplemental Fig. 2b) and Western blot analysis (supplemental Fig. 2c).

### Hoechst staining

BUMPT cells (generated by Drs. Wilfred Lieberthal and John Schwartz and obtained from Dr. Zheng Dong, Augusta University, Augusta, GA) were treated with cisplatin or/with sTim-3 protein (80 μg/mL) (with or without NF-κB inhibitors TPCA1 (50 μM) or PDTC (40 μM)) for 24 h. Apoptotic cells were analyzed by staining with Hoechst 33342 solution (10 μg/mL; Beyotime Biotechnology, China), and then observed with Nikon inverted microscope (NIKON A1R Storm) and calculated by ImageJ software.

### ROS examination

BUMPT cells were treated with cisplatin or/with sTim-3 protein (80 μg/mL) (with or without NF-κB inhibitors TPCA1 (50 μM) or PDTC (40 μM)) for 24 h. The serum-free medium was diluted with DCFH-DA (S0033S, Beyotime Biotechnology, China) with a concentration of 10 μmol/L, and then the DCFH-DA medium was used for incubation with BUMPT cells for 20 min at 37 °C. After washing three times with PBS, the cells were examined by flow cytometry (BD FACSCalibur).

### Immunofluorescence

BUMPT cells (1.5 × 10^5^ cells in 35 mm dishes) were treated with cisplatin or/with sTim-3 protein (80 μg/mL) for 24 h. After 24 h of treatment, the cells were incubated with cold methanol for about 10 min. The cells were treated with the primary anti‐8-OHdG antibody at 4 °C overnight and the secondary antibody conjugated with Alexa Fluor 488 for about 1 h and were counterstained with Hoechst 33342 (10 μg/mL; Beyotime company, China) for 5 min. The images were acquired by an inverted microscope (NIKON A1R Storm) and calculated by ImageJ software.

### Mitochondrial membrane potential examination

BUMPT cells were incubated with JC-1 working solution (1 mL, C2006, Beyotime company, China) for 20 min at 37 °C. After the incubation, cells were washed with JC-1 staining buffer and were then added with a 2 mL cell culture medium for observation under a confocal microscope (NIKON A1R Storm).

### Mitochondrial superoxide (MitoSOX) determination

BUMPT cells were treated with cisplatin or/with sTim-3 protein (80 μg/mL) for 18 h. Then, BUMPT cells were stained with MitoSOX Red dye (an indicator of mitochondrial superoxide, 5 μM, M36008, Thermo Fisher Scientific, USA) for 10 min at 37 °C, and then were observed using a confocal microscope (NIKON A1R Storm).

### Ca^2+^ signals examination

BUMPT cells (1.5 × 10^5^) were treated with cisplatin or/with sTim-3 protein (80 μg/mL) for 40 min. Then, BUMPT cells were stained with Fluo-4 AM working solution (5 μM, S1060, Beyotime Biotechnology, China) at 37 °C for 30 min. The green signals were examined using a confocal microscope (NIKON A1R STORM).

### Real-time reverse transcription PCR

RNA was extracted using Trizol (15596018, Thermo Fisher Scientific), and was reverse transcribed into cDNA using SYBR Select Master Mix kit (4472908, Thermo Fisher Scientific). The primers used for PCR were summarized in supplemental Table 3. qRT-PCR (quantitative reverse transcription–polymerase chain reaction) was performed with 7500 Fast PCR System (Thermo Fisher Scientific).

### Western blot analysis

BUMPT cells were stimulated with or without 40 μM cisplatin for about 24 h. SDS lysis buffer was used for protein extraction from BUMPT cells and kidney cortex tissues. Proteins were quantified by BCA quantification kit (CW0014, CWbiotech, China) and were separated by SDS–PAGE. After transferring to the PVDF membrane, proteins were incubated with primary antibodies overnight at 4 ^o^C and secondary antibodies for about 2 h. The primary antibodies were anti-Bax antibody (2772, CST), anti-caspase-3 (9662, CST), anti-Bcl-2 antibody (ab59348, Abcam), anti-Tim-3 antibody (ab185703, Abcam), anti-HO-1 antibody (52947, Abcam), anti-pIKKα/β (phosphor S176/180) antibody (2697, CST), anti-NF-κB p65 (phosphor S536) antibody (ab86299, Abcam), anti-PARP antibody (BS7047, Bioword), anti-8-OHDG antibody (sc-66036, Santa Cruz), and anti-GAPDH antibody (AC033, Abclonal). The second antibodies were an anti-rabbit IgG antibody (BA1054, Boster) and an anti-mouse IgG antibody (BA1050, Boster). Chemiluminescent signals were analyzed using an ECL detection kit (WBKLS0500; Millipore). The densities of bands were measured with ImageJ software.

### Statistical analysis

Quantitative variables were expressed as means ± standard deviation, and were analyzed by a researcher who was blinded. Comparison between groups were performed using one-way ANOVA or two-way ANOVA followed by Tukey’s post hoc test in Prism GraphPad v7.0 software. *p* values less than 0.05 are statistically significant.

### Supplementary information

Supplemental figure 1

Supplemental figure 2

Supplemental figure 3

Supplemental figure 4

Supplementary figure legends

Supplementary tables

Original Data File

## Data Availability

The data were available on request from the corresponding author.
