# Correction: Tim-3 protects against cisplatin nephrotoxicity by inhibiting NF-κB-mediated inflammation

**DOI:** 10.1038/s41420-023-01610-y

**Published:** 2023-09-06

**Authors:** Peiyao Li, Xuemiao Li, Wenbin Wu, Mengjia Hou, Guanyi Yin, Zhonghang Wang, Ziyu Du, Yuanfang Ma, Yinxiang Wei, Qiang Lou

**Affiliations:** Joint National Laboratory for Antibody Drug Engineering, The First Affiliated Hospital of Henan University, Henan University, Kaifeng, 475004 P.R. China

**Keywords:** Acute kidney injury, Apoptosis

Correction to: *Cell Death Discovery* 10.1038/s41420-023-01519-6, published online 01 July 2023

In this article the correspondence authors order is given erroneously. It should be read:

Qiang Lou, Yinxiang Wei

Also the order of the email address has been corrected to:

qiang_lou@126.com; yxwei@henu.edu.cn

The original article has been corrected.

