# Supplementary figures and images for: Tim-3 protects against cisplatin nephrotoxicity by inhibiting NF-κB-mediated inflammation
Source: Cell Death Discov. 2023 Jul 1;9:218. doi: 10.1038/s41420-023-01519-6 (PMC10314935; doi:10.1038/s41420-023-01519-6)

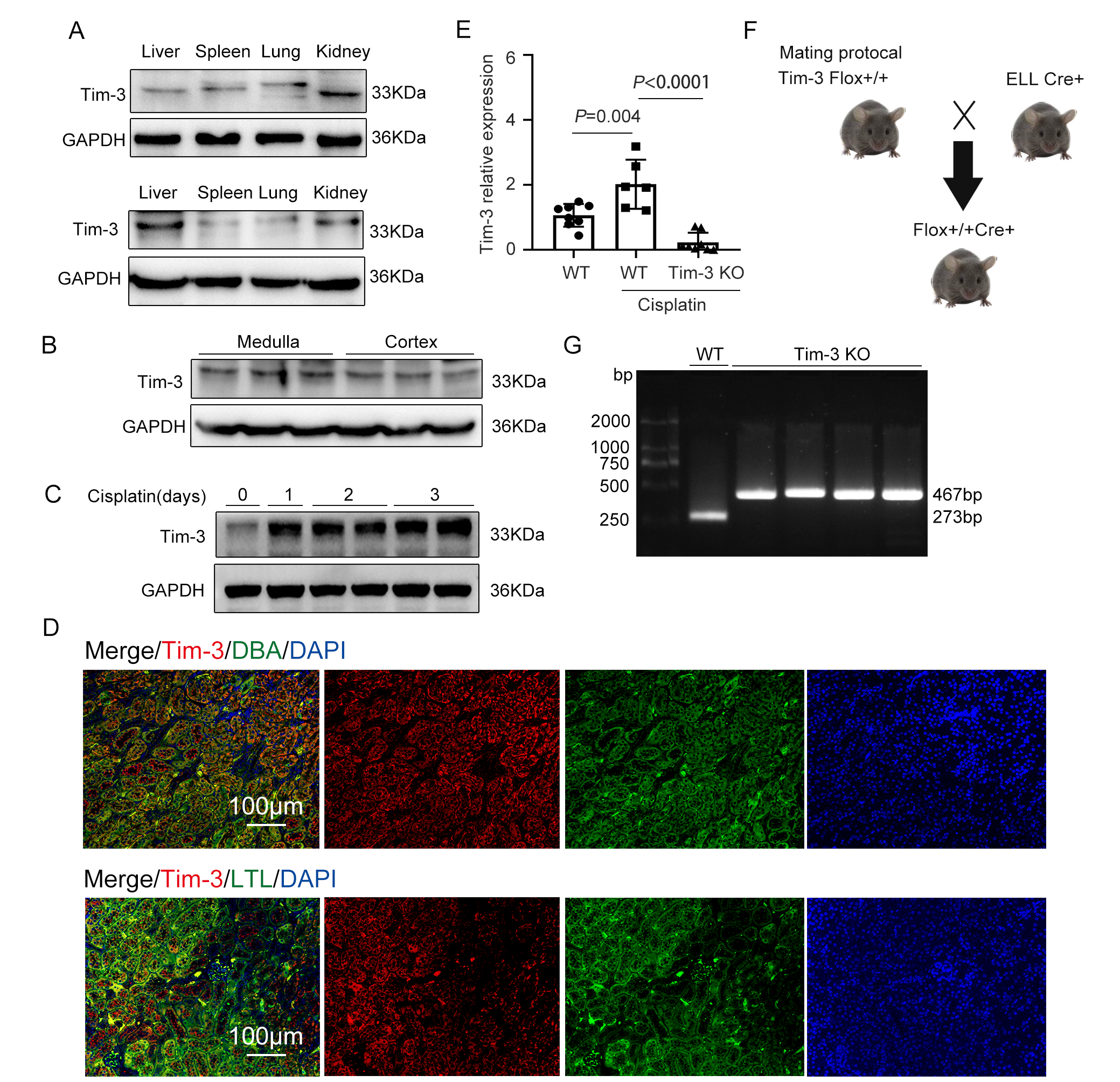

Supplement: Supplementary file 1 — Supplemental figure 1 [file 41420_2023_1519_MOESM1_ESM.tif]

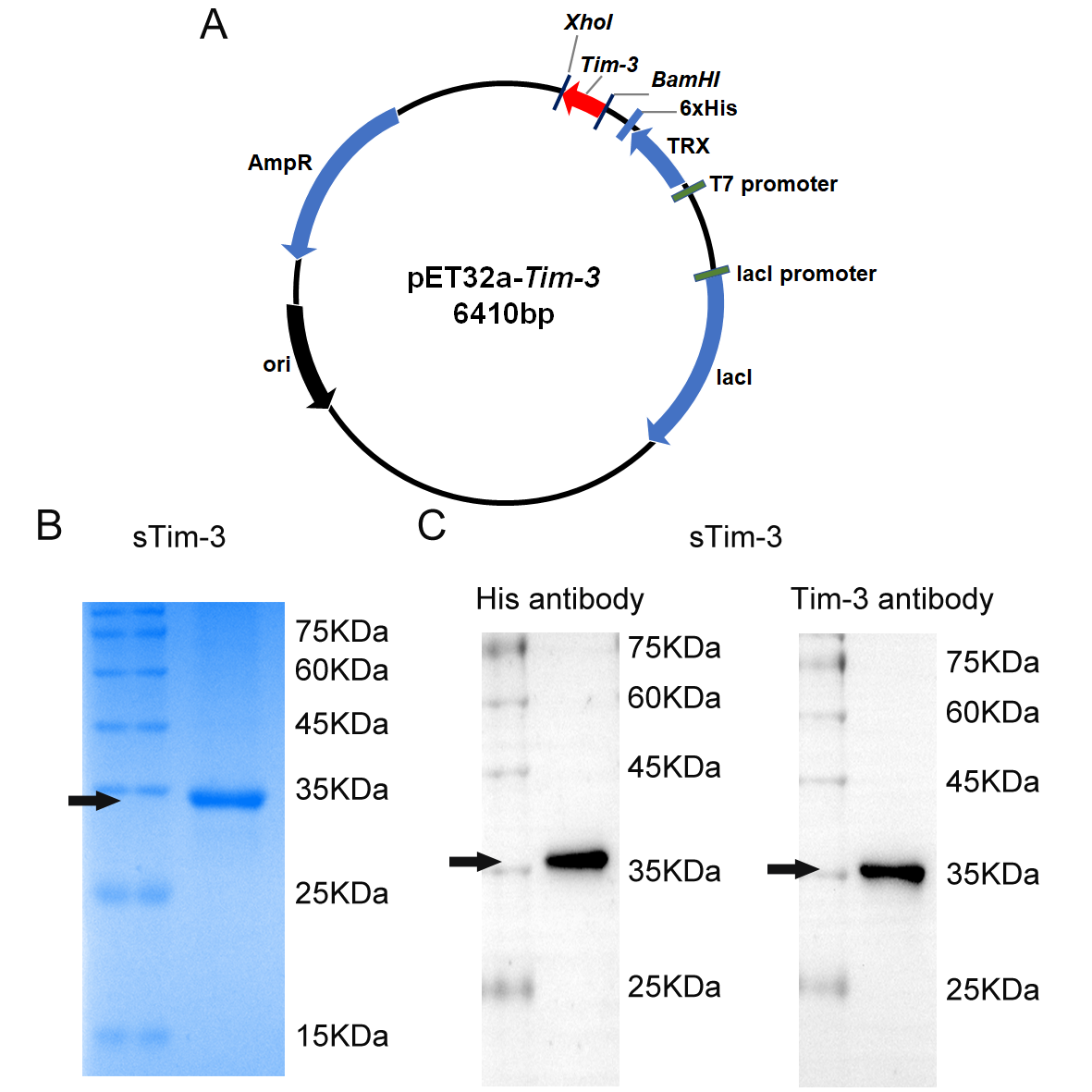

Supplement: Supplementary file 2 — Supplemental figure 2 [file 41420_2023_1519_MOESM2_ESM.tif]

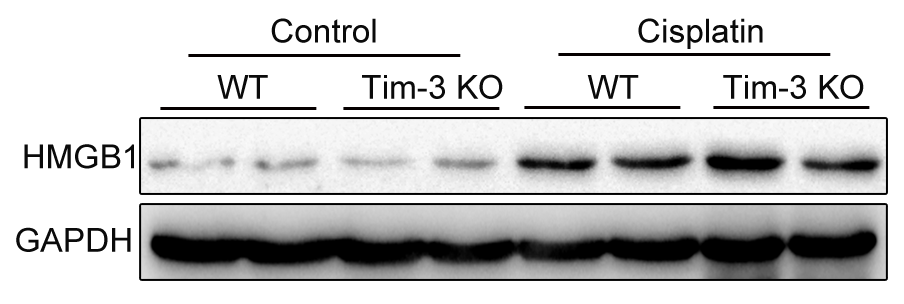

Supplement: Supplementary file 3 — Supplemental figure 3 [file 41420_2023_1519_MOESM3_ESM.tif]

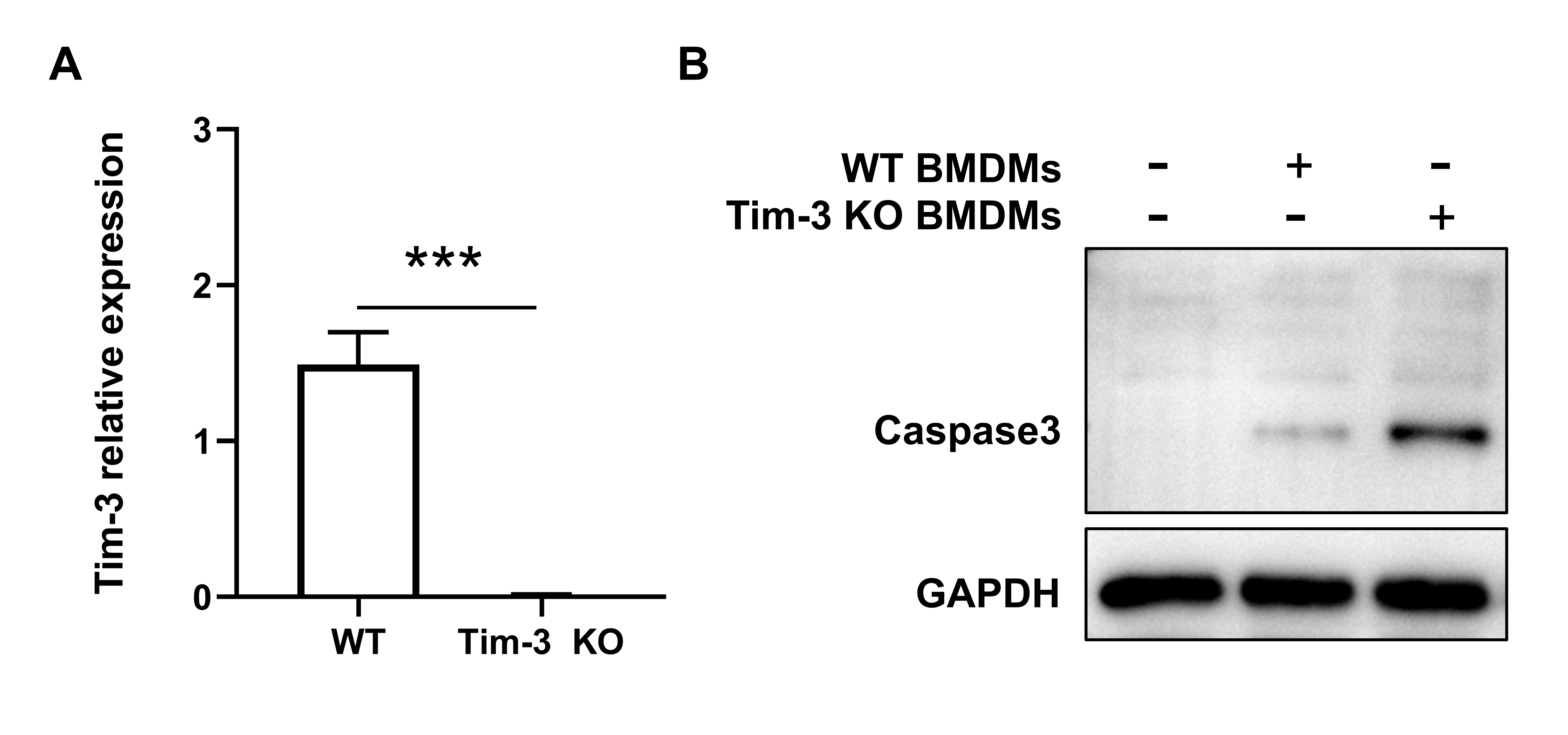

Supplement: Supplementary file 4 — Supplemental figure 4 [file 41420_2023_1519_MOESM4_ESM.tif]
