# Supplementary material for: Tim-3 protects against cisplatin nephrotoxicity by inhibiting NF-κB-mediated inflammation
Source: Cell Death Discov. 2023 Jul 1;9:218. doi: 10.1038/s41420-023-01519-6 (PMC10314935; doi:10.1038/s41420-023-01519-6)
Supplement: Supplementary file 5 — Supplementary figure legends [file 41420_2023_1519_MOESM5_ESM.docx]

**Fig.S1.** (A) Total proteins were extracted from kidney, liver, spleen and lung tissues, respectively. The expression of Tim-3 was determined by Western blot analysis. GAPDH was used as the internal control. The data of up panel and down panel are from two different mice. (B) Proteins were extracted from kidney medulla and kidney cortex sections. The expression of Tim-3 was determined by Western blot analysis. GAPDH was used as the internal control. (C) Western blot analysis of Tim-3 expression in kidney tissues after cisplatin treatment for 0, 1, 2 and 3 days. (D) Histoimmunofluorescence analysis of co-localization of Tim-3 and DBA/LTL in kidney tissue. (E) Real-time RT-PCR analysis of Tim-3 expression in kidney tissues of WT and Tim-3 KO mice with or without cisplatin treatment. (F) Tim-3-KO mice were generated via hybridization of Tim-3 Flox+/+ mice with EII Cre+ mice. (G) The genotyping assays were conducted by PCR of the tail venous serum of WT and Tim-3 knockout mice.

**Fig.S2.** (A) Plasmid map of recombinant vector pET32a-Tim-3. (B) SDS-PAGE of purified recombinant protein sTim-3 from *Escherichia coli* BL21 cells. sTim3 protein was purified after elution with 500 mM imidazole. (C) Western blot confirmation of purified recombinant protein sTim-3 (35 KD) using anti-His tag antibody and anti-Tim3 antibody, respectively.

**Fig.S3**. WT and Tim-3 KO mice were stimulated with cisplatin (30 mg/kg) for 72 hours. The expression of HMGB1 were determined by western blot analysis. GAPDH was used as the internal control.

**Fig.S4**. Effects of BMDMs supernatants on the apoptosis of BUMPT cells. Bone marrow-derived macrophages (BMDMs) were isolated from WT and Tim-3 KO mice. The BMDMs supernatants were further used to treat BUMPT cells for 12 hours. (A) Real-time RT-PCR analysis of Tim-3 expression in BMDMs from WT and Tim-3 KO mice. (B) Western blot analysis of the levels of cleaved caspase 3 of BUMPT cells.
