# Supplementary material for: Tim-3 protects against cisplatin nephrotoxicity by inhibiting NF-κB-mediated inflammation
Source: Cell Death Discov. 2023 Jul 1;9:218. doi: 10.1038/s41420-023-01519-6 (PMC10314935; doi:10.1038/s41420-023-01519-6)
Supplement: Supplementary file 6 — Supplementary tables [file 41420_2023_1519_MOESM6_ESM.docx]

**Table S1. List of primers used for genotyping.**

| Gene Name | Primers (5′-3′) |
| --- | --- |
| mHavcr2-Forward | CCAATTGGGTTCTACTATAAAGCCTTG |
| mHavcr2-Reverse1 | AAGTTGAGAGTTCTGGGATTACAGG |
| mHavcr2-Reverse2 | ATACTTGCTTCAGTGGCTCGCGA |

**Table S2. Primers used for PCR amplification of mouse Tim-3 extracellular domain (134-643 bp).**

| Gene Name | Primers (5′-3′) |
| --- | --- |
| upstream | ATCGGGATCCTTGGAAAATGCTTATGTGTTTGAGGTT |
| downstream | GCTACTCGAGTTATCATCTGATCGTTTCTCCAGAGTCCTT |

**Table S3. List of primers used for real-time PCR.**

| Gene Name | Forward (5′-3′) | Reverse (5′-3) |
| --- | --- | --- |
| IL-1β | GAAATGCCACCTTTTGACAGTG | CTGGATGCTCTCATCAGGACA |
| TNF-α | CTGGGACAGTGACCTGGACT | GCACCTCAGGGAAGAGTCTG |
| IL-10 | AAGGCAGTGGAGCAGGTGAAGA | TGGTGAGTAGACAGAGGTCTTGTTACA |
| Tim-3 | CAGGTCTTACCCTCAACTGTG | GGCAGATAGGCATTTTTACCA |
| GAPDH | AGGTCGGTGTGAACGGATTTG | TGTAGACCATGTAGTTGAGGTCA |
