# Supplementary material for: Tim-3 protects against cisplatin nephrotoxicity by inhibiting NF-κB-mediated inflammation
Source: Cell Death Discov. 2023 Jul 1;9:218. doi: 10.1038/s41420-023-01519-6 (PMC10314935; doi:10.1038/s41420-023-01519-6)

Figure 1A

GAPDH:

35KDa

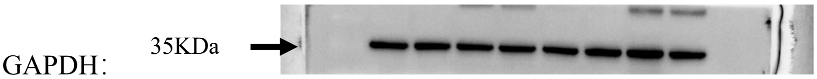

Tim-3:

35KDa

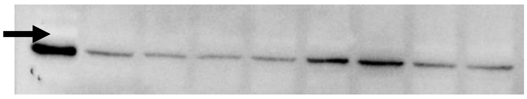

HO-1:

35KDa

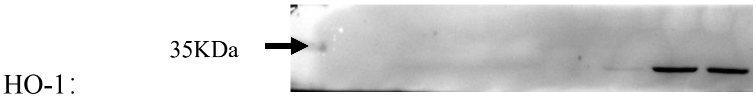

Bax:

25KDa

15KDa

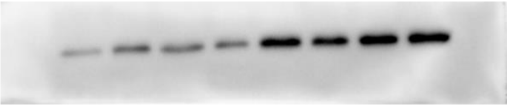

Figure 2A

Tim-3:

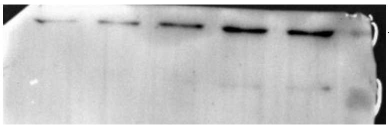

35KDa

25KDa

Caspase3:

35KDa

25KDa

15KDa

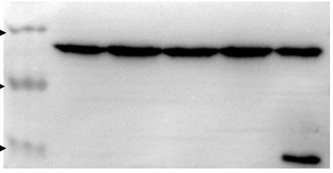

GAPDH:

40KDa

35KD

25KDa

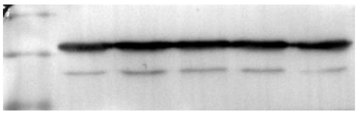

Figure 2D

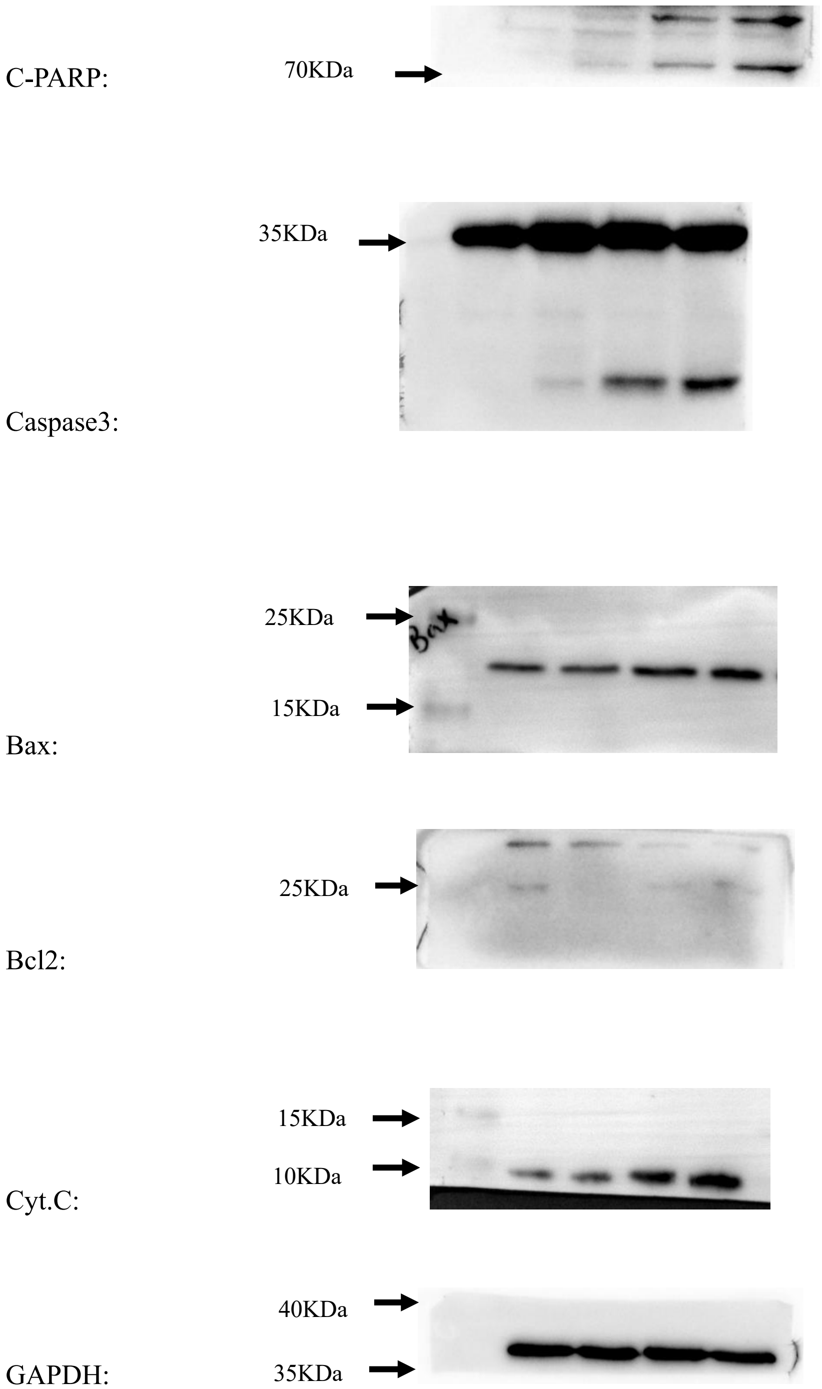
C-PARP:
70KDa
35KDa
Caspase3:
25KDa
15KDa
Bax:
25KDa
Bcl2:
15KDa
10KDa
Cyt.C:
40KDa
35KDa
GAPDH:

Figure 5B

GAPDH:

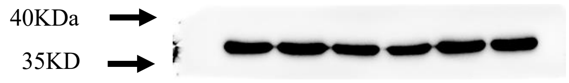

P65:

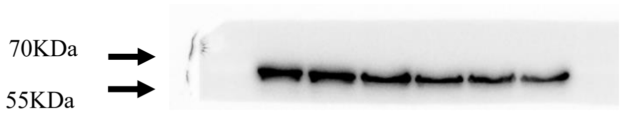

p-P65:

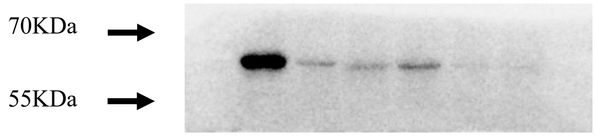

Caspse3:

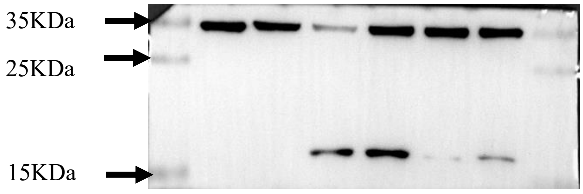

Figure 5F

Caspase3:

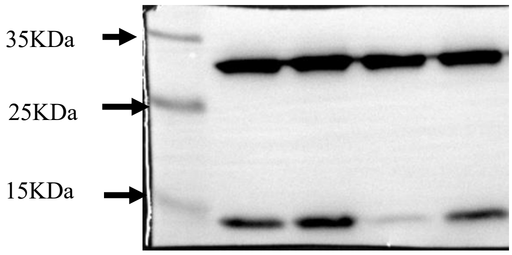

GAPDH:

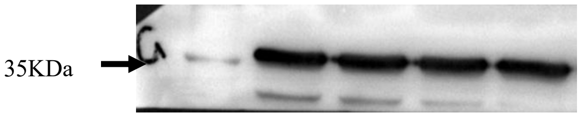

Supplemental figure 1A

GAPDH:

35KDa

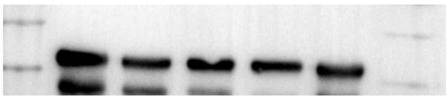

Tim-3:

35KDa

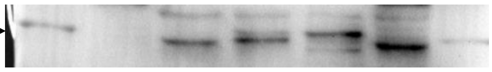

GAPDH:

35KDa

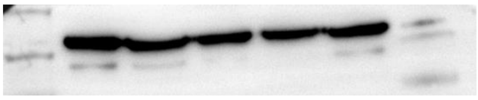

Tim-3

35KDa

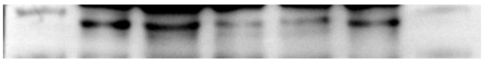

GAPDH:

35KDa

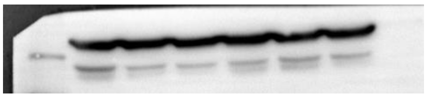

Tim-3:

35KDa

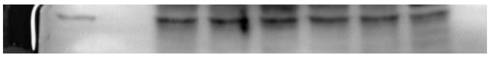

Supplemental figure 2A

Tim-3:

75KDa
60KDa
45KDa
35KDa
25KDa

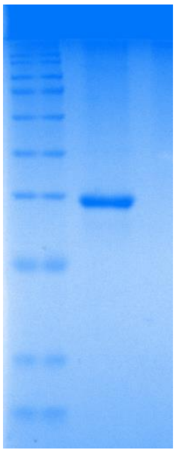

Supplemental figure 2B

His:

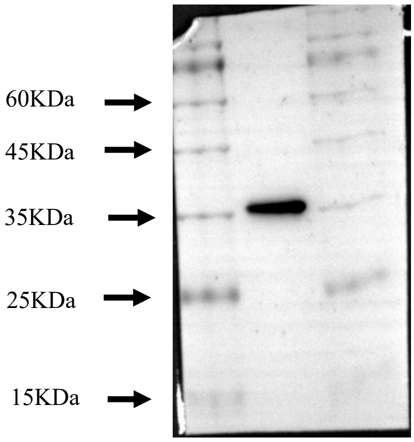

Tim-3:

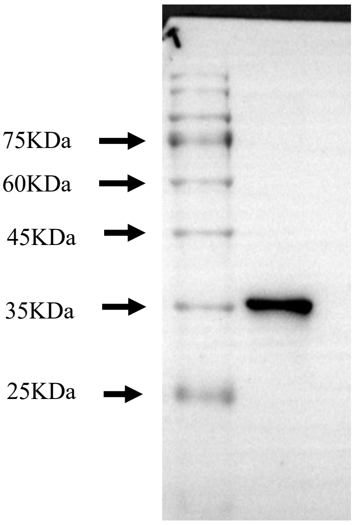

Supplemental figure 3

HMGB1:

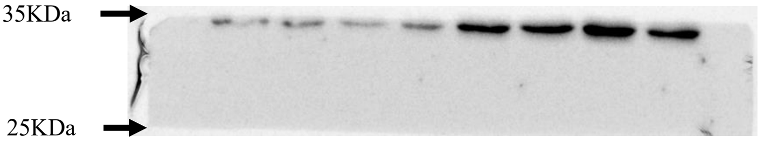

GAPDH:

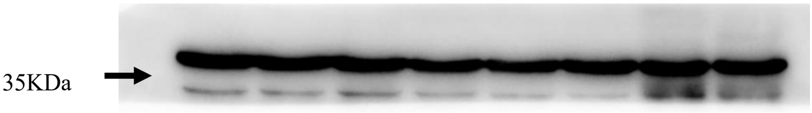

Supplemental figure 4

Caspase3：

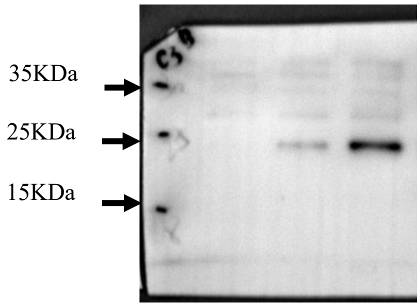

GAPDH:

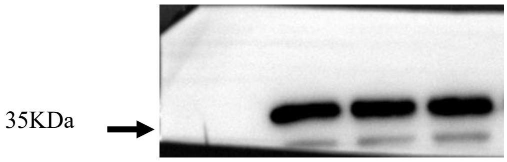

Supplement: Supplementary file 7 — Original Data File [file 41420_2023_1519_MOESM7_ESM.pdf]
